# Deficiency of liver-derived insulin-like growth factor-I (IGF-I) does not interfere with the skin wound healing rate

**DOI:** 10.1371/journal.pone.0193084

**Published:** 2018-03-13

**Authors:** Ileana Ruxandra Botusan, Xiaowei Zheng, Sampath Narayanan, Jacob Grünler, Vivekananda Gupta Sunkari, Freja S. Calissendorff, Ishrath Ansurudeen, Christopher Illies, Johan Svensson, John-Olov Jansson, Claes Ohlsson, Kerstin Brismar, Sergiu-Bogdan Catrina

**Affiliations:** 1 Department of Molecular Medicine and Surgery, Karolinska Institutet, Solna, Stockholm, Sweden; 2 Department of Endocrinology, Diabetes and Metabolism, Karolinska Institutet, Karolinska University Hospital, Stockholm, Sweden; 3 Center for Diabetes, Academic Specialist Center, Stockholm County Council, Sweden; 4 Department of Clinical Pathology, Karolinska Institutet, Karolinska University Hospital, Stockholm, Sweden; 5 Institute of Internal Medicine, Sahlgrenska Academy, University of Gothenburg, Göteborg, Sweden; 6 Institute of Neuroscience and Physiology, Sahlgrenska Academy, University of Gothenburg, Göteborg, Sweden; University of Alabama at Birmingham, UNITED STATES

## Abstract

**Objective:**

IGF-I is a growth factor, which is expressed in virtually all tissues. The circulating IGF-I is however derived mainly from the liver. IGF-I promotes wound healing and its levels are decreased in wounds with low regenerative potential such as diabetic wounds. However, the contribution of circulating IGF-I to wound healing is unknown.

Here we investigated the role of systemic IGF-I on wound healing rate in mice with deficiency of liver-derived IGF-I (LI-IGF-I-/- mice) during normal (normoglycemic) and impaired wound healing (diabetes).

**Methods:**

LI-IGF-I-/- mice with complete inactivation of the IGF-I gene in the hepatocytes were generated using the Cre/loxP recombination system. This resulted in a 75% reduction of circulating IGF-I. Diabetes was induced with streptozocin in both LI-IGF-I-/- and control mice. Wounds were made on the dorsum of the mice, and the wound healing rate and histology were evaluated. Serum IGF-I and GH were measured by RIA and ELISA respectively. The expression of IGF-I, IGF-II and the IGF-I receptor in the skin were evaluated by qRT-PCR. The local IGF-I protein expression in different cell types of the wounds during wound healing process was analyzed using immunohistochemistry.

**Results:**

The wound healing rate was similar in LI-IGF-I-/- mice to that in controls. Diabetes significantly delayed the wound healing rate in both LI-IGF-I-/- and control mice. However, no significant difference was observed between diabetic animals with normal or reduced hepatic IGF-I production. The gene expression of IGF-I, IGF-II and IGF-I receptor in skin was not different between any group of animals tested. Local IGF-I levels in the wounds were similar between of LI-IGF-I-/- and WT mice although a transient reduction of IGF-I expression in leukocytes in the wounds of LI-IGF-I-/- was observed seven days post wounding.

**Conclusion:**

Deficiency in the liver-derived IGF-I does not affect wound healing in mice, neither in normoglycemic conditions nor in diabetes.

## Introduction

IGF-I has growth promoting effects and it is expressed in virtually all tissues [1]. The circulating IGF-I is however derived mainly from the liver. Canonically, the longitudinal body growth was considered to be dependent on the IGF-I produced in the liver under the control of growth hormone (GH) [2]. However this theory was challenged by the finding that mice lacking the liver IGF-I secretion have unaffected postnatal body growth [3–5]. Moreover, a local secretion of IGF-I was demonstrated in multiple tissues [6].

Beyond the key role as body growth regulator, IGF-I plays critical roles to maintain the normal function of several organs e.g. kidneys, cardiovascular system, and brain [7]. Moreover, IGF-I promotes wound healing by multiple mechanisms as it has chemotactic activity for endothelial cells, it stimulates the proliferation of keratinocytes and fibroblasts, and it increases the wound strength [8].

However, the relative contribution of the liver-derived IGF-I (endocrine- acting) versus the locally produced IGF-I (autocrine- paracrine acting) for wound healing is still unknown. Even clinical situations with characteristic abnormal wound healing as diabetes [9] do not help in understanding the contribution of different sources of IGF-I for wound healing since both serum and local wound IGF-I expression are reduced in diabetes [8, 10].

We have therefore proposed to investigate in this study the role of systemic IGF-I on cutaneous wound healing using liver-specific IGF-I deficient (LI-IGF-I-/-) mice that have a 75% reduction of hepatic IGF-I production. The study was performed not only in normoglycemic mice but also in diabetic mice where wound healing is known to be impaired by hyperglycemia [11].

## Materials and methods

### Animals

Mice with complete inactivation of the IGF-I gene in hepatocytes (LI-IGF-I-/- mice) were generated as previously described [3]. Briefly, Mx-Cre 31 mice were crossed with mice with exon 4 of the IGF-I gene flanked with loxP sites. Mice homozygous for loxP and positive for Mx-Cre expression were used as LI-IGF-I-/- mice and mice homozygous for loxP but lacking Mx-Cre expression were used as controls. Both LI-IGF-I-/- and control mice were treated at 1 month of age with interferon which activated Mx-Cre 31. The experimental procedure was approved by the North Stockholm Ethical Committee for Care and Use of Laboratory Animals.

### Streptozocin-induced diabetes

Diabetes was induced in 2 months old LI-IGF-I-/- *mice* and in their controls using streptozotocin (STZ) according to the instructions from AMDCC (*Animal Models of Diabetic Complications Consortium*) as previously described [11]. Animals were kept diabetic for three weeks before the start of wound healing experiment.

### Wound model

The wound model was made as previously described [12]. Briefly, two full-thickness wounds extending through the panniculus carnosus were made on the dorsum on each side of midline using a 6 mm biopsy punch after blood glucose was controlled and after induction of general anaesthesia. Isofluran mask was used for anaesthesia induction and analgesia was further controlled with buprenorphine (Temgesic). Digital photographs were recorded at the day of surgery and every other day after wounding. A circular reference was placed alongside of the animals to permit the correction for the distance between camera and animals. The wound area was calculated using the Image J 1.32 (N.I.H., U.S.A.) software, corrected for the area of the reference circle and expressed as percentage of the original area. The investigators were blinded to the identity of the mice during wound area analysis. Wounds were harvested at the end of the experiment for histology analysis and mRNA expression. One wound was snap-frozen in liquid nitrogen and the other one was fixed in 4% paraformaldehyde in PBS.

### Histology and immunohistology

Histological analysis was performed on formalin-fixed, paraffin-embedded sections (5μM). After deparaffinisation and rehydration the slides were stained with hematoxylin and eosin and examined by two independent observers unaware of the identity of the biopsy. A four-point scale was used to evaluate the granulation tissue formation (1, thin granulation layer; 2, moderate granulation layer; 3, thick granulation layer; and 4, very thick granulation layer) [13].

Collagen staining and quantification: the slides were deparaffinised as above then stained with Weigert’s hematoxylin, tungstophosphoric acid orange G solution and light green SF, and rinsed in 1% acetric acid between each of the staining steps according to the manufacturer protocol of Masson Goldner staining kit from Merck-Millipore (Cat # 1004850001). The images were acquired using Leica DM3000 LED fluorescence microscope. Image analysis was performed using Image-Pro Premier software (Media cybernetics). Three-five images of the wounds from each condition were analysed. The in-built smart segmentation option was used to differentiate collagen stained, unstained and nuclear regions. Collagen staining is expressed as the percentage of the area stained by collagen to total area.

### Fluorescent immunohistochemistry

The frozen tissue sections were fixed sequentially with 50% acetone for 30 seconds and 100% acetone for 5 minutes. The slides were then washed with PBS-T (0.1% tween) 3 times for 3 minutes each. The sections were blocked with goat serum or 5% BSA in PBS for 30 minutes at RT, then incubated with primary antibodies overnight at 4°C. After 3 times wash with PBS-T for 3 minutes each, the sections were incubated with fluochrome-conjugated secondary antibody for one hour at RT in dark. After washing, the slides were treated with 1 ug/mL DAPI (Life Technologies) in PBS for 5 minutes at RT. The sections were then mounted and stored in 4°C. The fluorescent images were acquired using a laser scanning confocal microscope (LSM 510, Carl Zeiss, Germany). Image analysis was performed using ImageJ v1.47 software. Four to seven fields from each slide were analysed, at least 3 slides for each condition. The IGF-I, CD31 or CD11b positive cells were expressed as ratio from the total cell numbers stained by DAPI. Double positive stained cells IGF-I/Cd11b or IGF-I/CD31 were expressed relative to the total cell number counted by DAPI-positive cells. Primary antibodies: Rat monoclonal anti-CD31 antibody (Dianova, DIA-310), rat monoclonal anti-CD11b antibody (EMD Millipore, MABF512), and rabbit polyclonal anti-IGF-I antibody (Abcam, ab40657).Secondary antibody: Goat anti-rabbit IgG, Alexa Fluor 594 (A-11037, ThermoFisher Scientific; 1:500), goat anti-rat Alexa-Fluor 488 (A-11006, ThermoFisher Scientific; 1:500).

### Serum assays

The serum IGF-I levels were measured by RIA after acid–alcohol extraction and cryoprecipitation as described [14]. The sensitivity of the RIA was 6 μg/L and the intra- and inter-assay coefficients of variations were 4 and 11%, respectively.

The serum Growth Hormone (GH) was measured by ELISA using Mouse Growth Hormone (Millipore), according with the manufacturer instructions. The sensitivity of the ELISA kit was 0.07 ng/μL and the intra- and inter-assay coefficients of variations were 4 and 4.9%, respectively.

### Real-time RT-PCR

Total RNA was isolated from skin using RNeasy Fibrous Tissue Mini Kit (Qiagen). cDNA was obtained using High-Capacity cDNA Reverse Transcription Kit (ThermoFisher Scientific). Real-time PCR was performed on 7300 or 7900 Real-Time PCR System (Applied Biosystems) by Taqman gene expression assay. The mRNA expression level of PBGD (porphobilinogen deaminase) was used as internal control. The Taqman gene expression assays (Assay ID Mm00439560_m1 for IGF-I, Mm00439564_m1 for IGF-II, Mm00802831_m1 for IGF-1R, Mm01143545_m1 for PBGD) and Taqman Universal Mater Mix (Catalog number: 4440046) were obtained from ThermoFisher Scientific. The real-time PCR reaction includes 40 cycles of 95°C for 15 sec followed by 60 degree for 1 min

### Statistical analysis

Differences between groups were computed using Student t-test, one-way analysis or two-way repeated measures of variance (ANOVA) as appropriate, with *Bonfferoni* post hoc test. Outliers were detected by Grubbs’ test. A *p* <0.05 was considered statistically significant. The results were presented as mean ± S.E.M.

## Results

### Decrease of the circulating IGF-I by liver-specific IGF-I inactivation does not affect the wound healing rate

**Fig 1:**
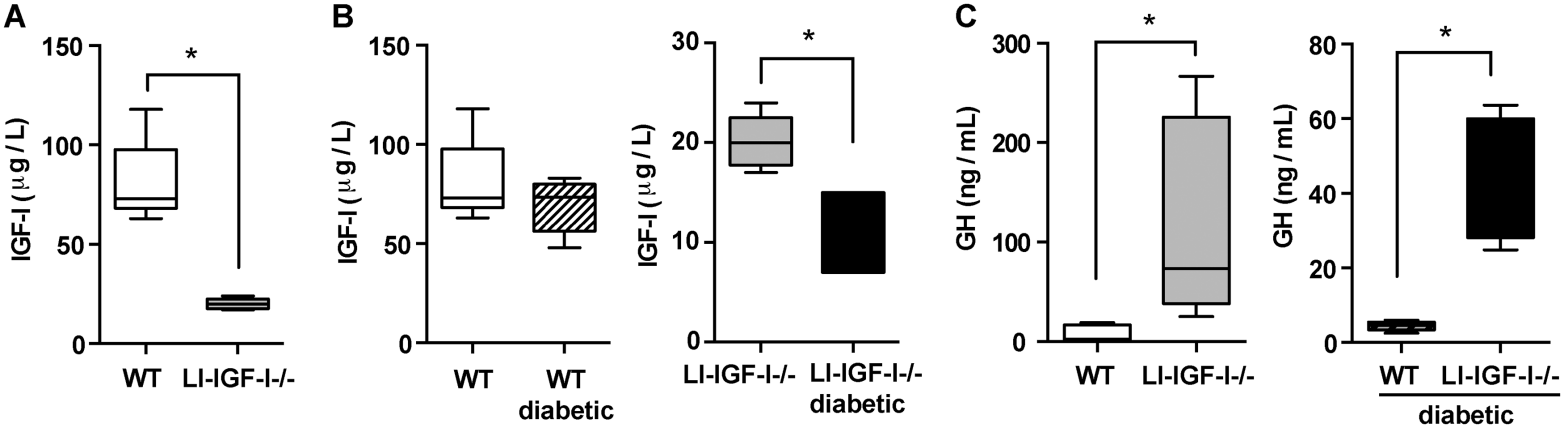
Serum IGF-I and GH levels in liver-specific IGF-I deficient mice versus controls. **(A)** Serum IGF-I values in liver-specific IGF-I deficient mice (LI-IGF-I-/-) and wild-type (WT) control mice. *P< 0.05. **(B)** Serum IGF-I levels in diabetic and normoglycemic mice. *Left panel*: WT control mice; *Right panel*: LI-IGF-I-/- mice.* *P*<0.05 between normoglycemic versus diabetic LI-IGF-I -/- mice. **(C)** serum GH values in normoglycemic (left panel) and diabetic (right panel) LI-IGF-I-/- and WT control mice. * *P*<0.05.

**Fig 2:**
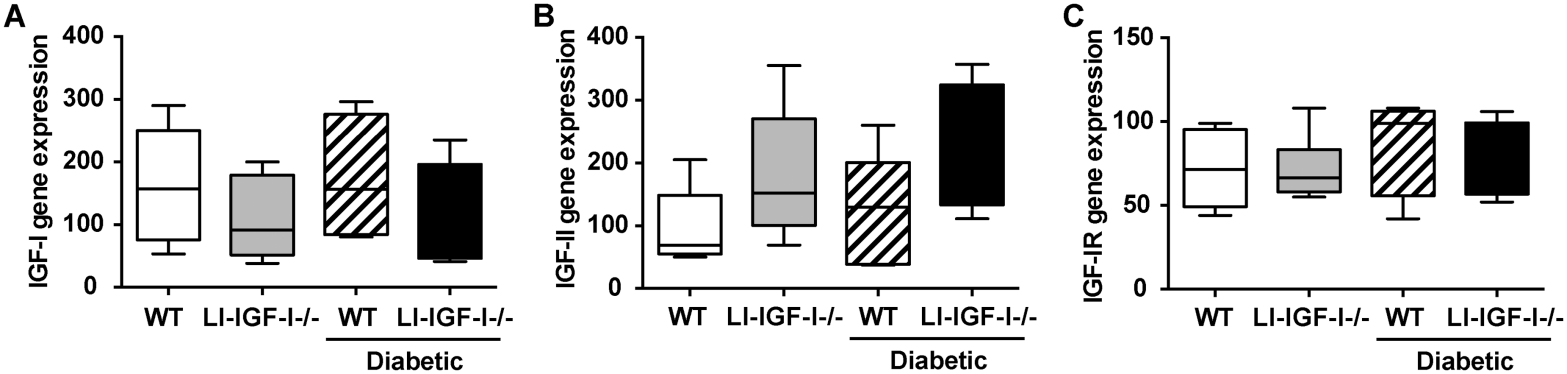
The mRNA expression of IGF-I, IGF-II and IGF-IR in the skin of liver-specific IGF-I deficient mice versus WT control animals with or without diabetes.

**Fig 3:**
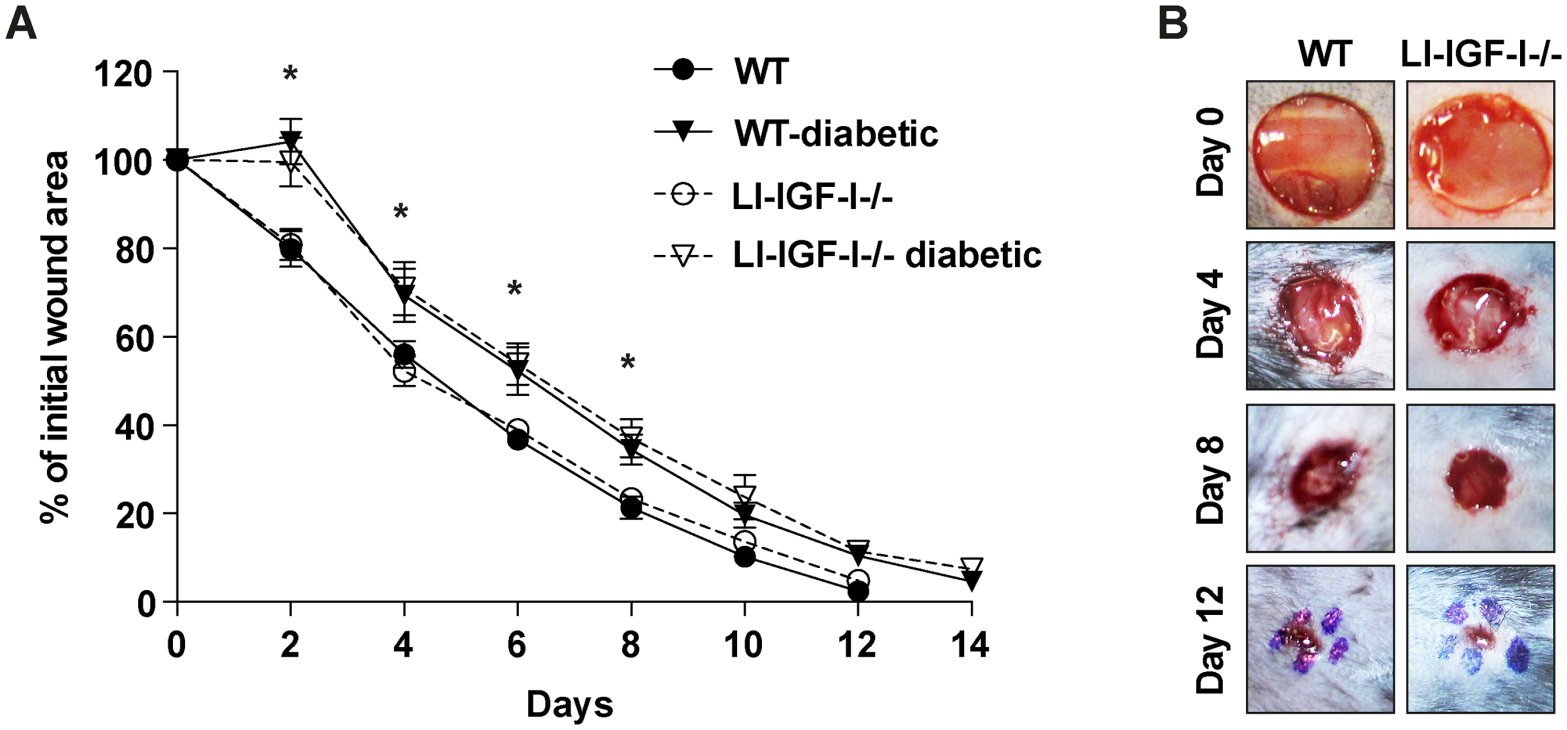
Deficiency of liver-derived IGF-I does not interfere with cutaneous wound healing rate. **(A)** The wound healing rate of full thickness wounds is similar in LI-IGF-I-/- mice compared with their normal IGF-I secreting controls both in normoglycemic and in diabetic conditions.*P< 0.05 between diabetic mice versus normoglycemic mice. **(B)** Representative wound images of the given time points during the healing process in normoglycemic LI-IGF-I-/- mice versus WT control.

Inactivation of IGF-I gene in the liver of LI-IGF-I-/-mice resulted in a 75% reduction of serum IGF-I (Fig 1A). Diabetes decreased even further the serum levels of IGF-I in LI-IGF-I-/-mice (Fig 1B). As expected, the levels of serum GH increased in LI-IGF-I-/- mice secondary to the decrease in serum IGF-I (Fig 1C). The decrease of the circulating IGF-I and the compensatory increase in GH were not followed by significant changes in the expression of IGF-I, IGF-II and IGF-I receptor (IGF-IR) in the skin (Fig 2). Despite lower levels of serum IGF-I, the wound healing rate in the LI-IGF-I-/- mice was similar to the wild-type (WT) control mice (50% wound closure at 4 +/- 0.5 days versus 4 +/- 0.3 days,) (Fig 3).

Moreover we investigated the relative contribution of the liver-derived IGF-I for the wound healing even in a pathological situation, as diabetes with characteristically impaired wound healing rate. The glucose levels of the diabetic animals at the start of the wound healing experiment (3 weeks after the induction of diabetes) were 19.14±1.36 mmol/l in the LI-IGF-I-/- group and 19.43±2.06 mmol/l in the WT-diabetic group. Diabetes delayed as expected the wound healing rate compared with normoglycemic animals but no difference was observed between the diabetic LI-IGF-I-/- and diabetic WT control mice (50% wound closure at 6 +/- 0.1 days versus 6 +/- 0.2 days) (Fig 3).

### Central processes involved in wound healing are similar in LI-IGF-I-/- mice versus WT control

Central processes involved in wound healing were analyzed in LI-IGF-I-/- mice versus WT control after hematoxylin eosin (HE) staining (S1 Fig).

**Fig 4:**
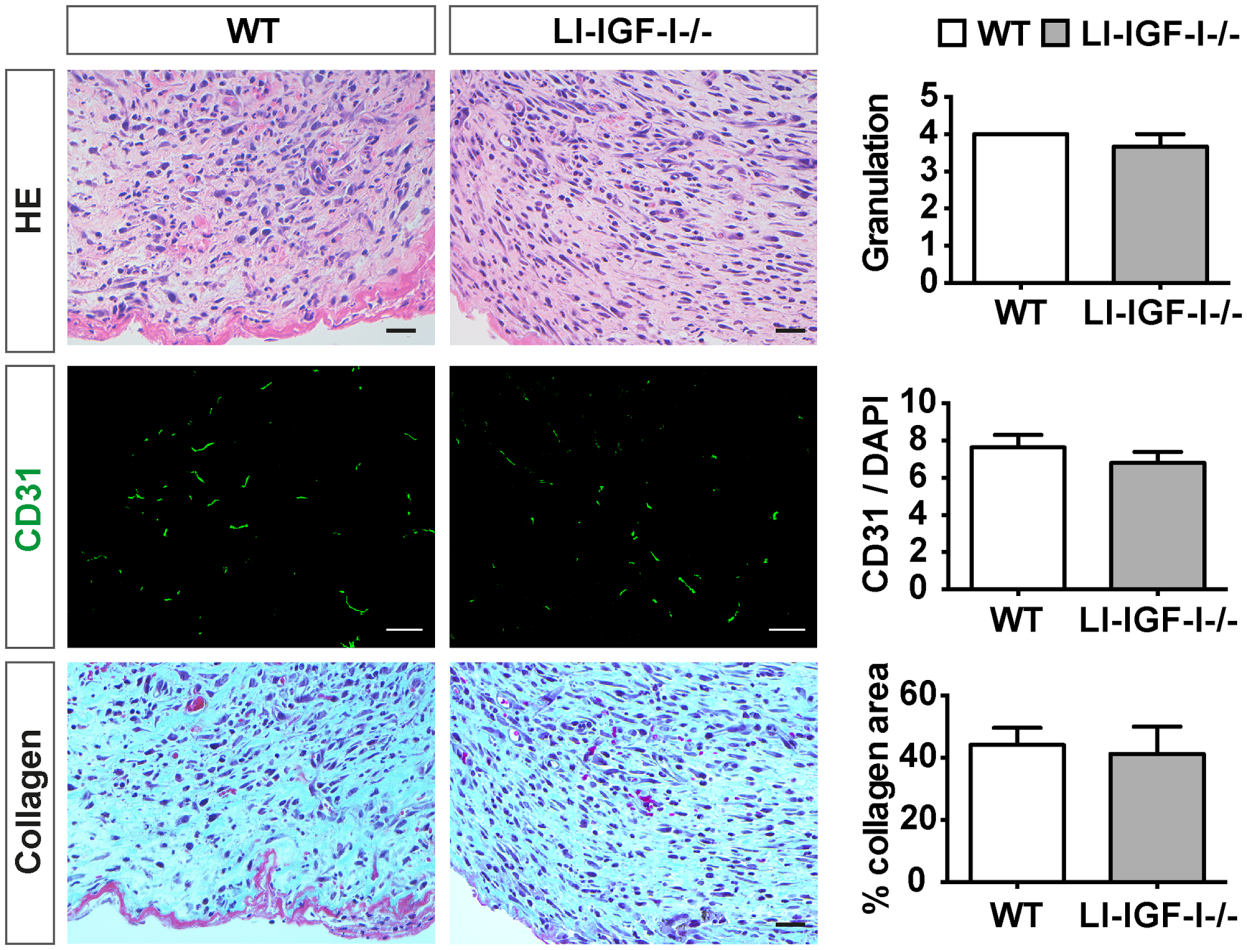
Histological analysis of the wounds in liver-specific IGF-I deficient mice versus controls. Granulation and collagen deposition level were evaluated by histological analysis after hematoxylin-eosin (HE) and Masson Goldner staining, respectively (scale bar: 25 μm). Angiogenesis was analyzed after anti-CD31 immunohistochemical staining (scale bar: 100 μm).

The rate of granulation tissue formation was similar in LI-IGF-I-/- mice and WT controls (Fig 4A), and there was no significant difference in angiogenesis (Fig 4B) or collagen deposition (Fig 4C) confirming a similar healing rate between the two groups.

**Fig 5:**
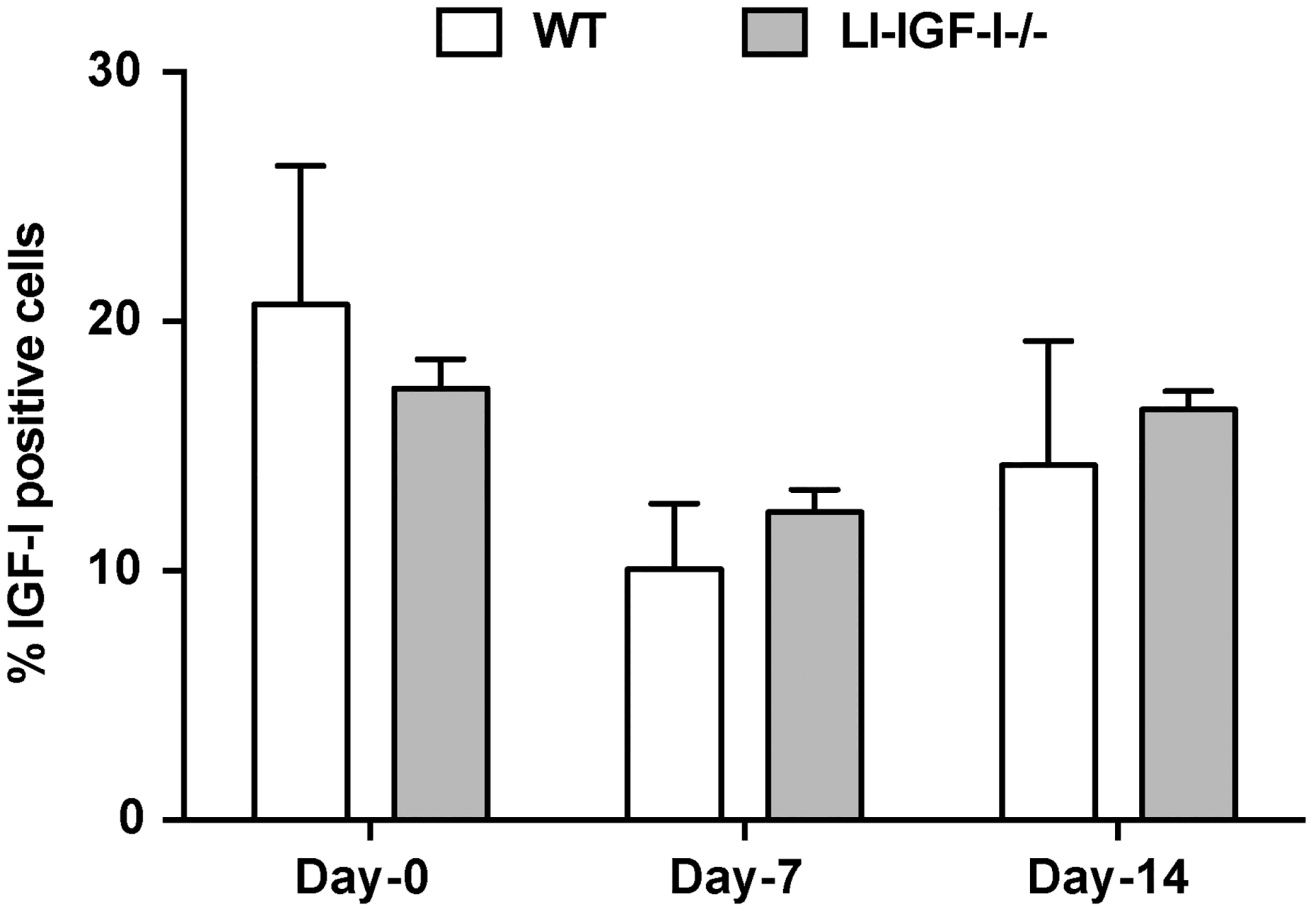
IGF-I expression analysis in the tissues during wound healing process. After immunohistochemical staining of IGF-I in the tissues from wounding day (Day-0) and after 7 days (Day-7) and 14 days (Day-14) post wounding, the percentage of IGF-I positive cells were evaluated.

Because the circulating IGF-I levels were significantly different between WT and LI-IGF-I-/- mice, we further analyzed IGF-I levels in the wounds during healing process. As shown in Fig 5, the presence of IGF-I in the wounds did not differ between WT control and LI-IGF-I-/- mice at any time point assessed (wounding day, 7 days post wounding and at the end of the experiment).

**Fig 6:**
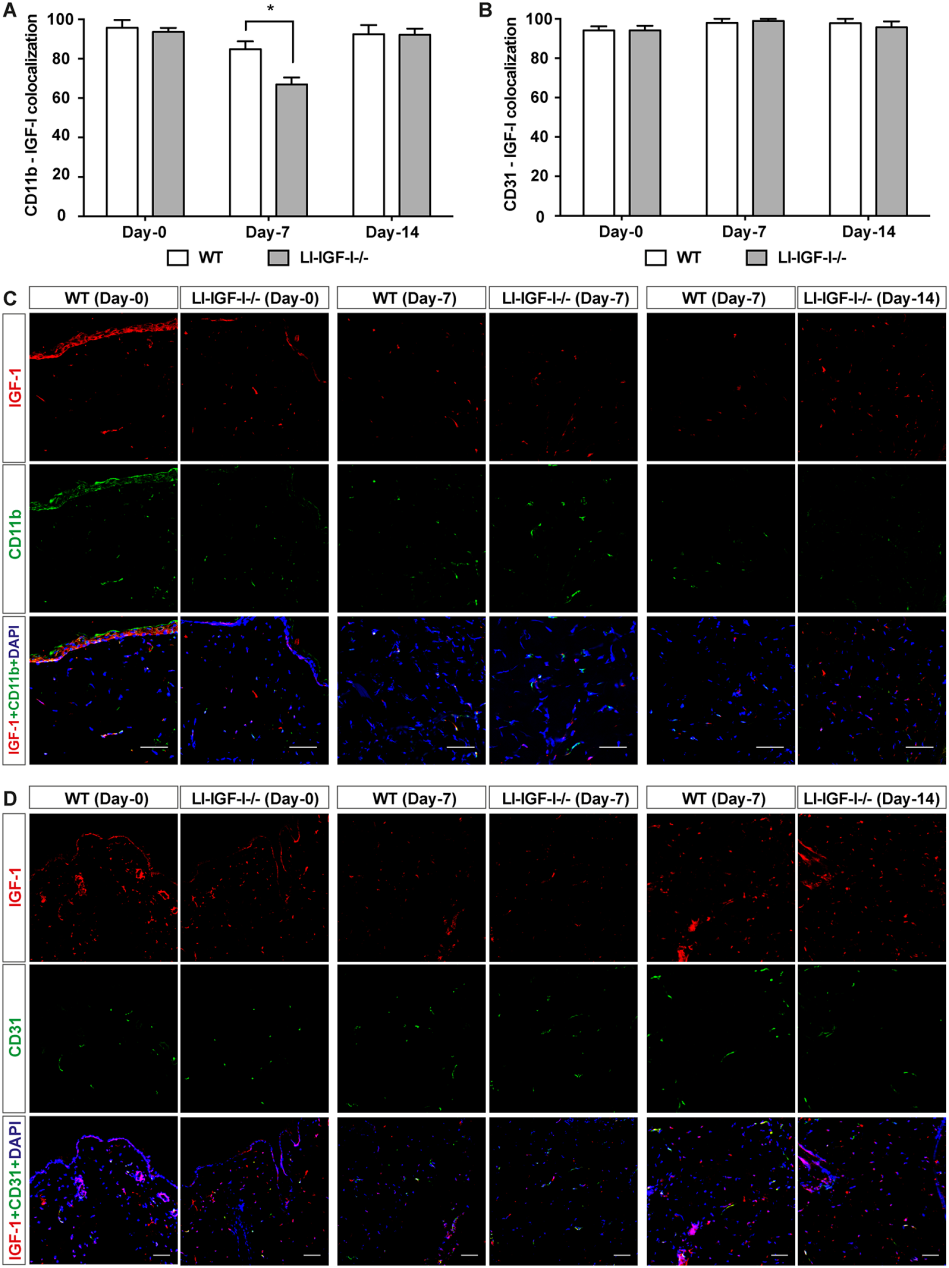
Cellular IGF-I expression during wound healing process. Tissue sections from WT and liver-specific IGF-I deficient (LI-IGF-I-/-) mice on wounding day (Day-0) and after 7 days (Day-7) or 14 days (Day-14) post wounding were double-stained with IGF-I and CD11b or CD31 antibody. IGF-I expression in leukocytes **(A and C)** or endothelial cells **(B and D)** were evaluated as percentage of IGF-I-positive cells in CD11b-positive or CD31-positive cells, respectively. Nuclei were counterstained with DAPI. Scale bar: 100 μm.

However, the Mx-cre 31 interferon inducible model used for generating the LI-IGF-I-/- mice could alter, apart from the targeted hepatocytes, IGF-I expression in leukocytes such as macrophages and other cells involved in wound healing. We therefore further evaluated the IGF-I distribution in different cell types that are important for wound healing. This was done by the analysis of the co-localized IGF-I expression in CD31-postive endothelial cells, in CD11b-positive leukocytes including granulocytes, monocytes, macrophages, and lymphocytes as well as in keratinocyte layer (Fig 6). Indeed we observed significant lower IGF-I expression in CD11b-positive leukocytes from LI-IGF-I-/- mice compared with their WT controls seven days post wounding while no difference was noticed at the beginning or at the end of the experiment (Fig 6A and 6C). The CD11b-positive leukocyte cell number was not different between LI-IGF-I-/- and WT mice (S2 Fig). No difference was observed between the WT and LI-IGF-I-/- mice regarding IGF-I distribution either in CD31-positive endothelial cells (Fig 6B and 6D) or in keratinocyte layer which showed IGF-I positivity in all cells (Fig 6).

## Discussion

Here we have addressed the importance of systemic, liver-derived IGF-I for cutaneous wound healing processes. We show that liver-specific IGF-I inactivation does not affect the wound healing, neither under normoglycemic conditions nor in diabetes. Regarding the important role played by IGF-I for wound healing [8], our data showed that even a 75% reduction of serum IGF-I is not sufficient to decrease enough IGF-I availability at the wound site to affect the normal healing rate. This is not unique for wound healing since in the same mouse model, the same levels of serum IGF-I levels are enough to sustain an appropriate body growth [3, 4].

The “minimum serum IGF-I level” sufficient for preserving the normal body growth was proposed to be in the range of 10–25% of normal serum IGF-I levels as suggested by several genetic manipulated animal models [7, 15]. Our data confirm that the same range of serum IGF-I levels are sufficient for preserving normal wound healing rate as well. In the condition of lower circulating IGF-I levels GH secretion is compensatory increased due to lack of IGF-I-mediated negative feedback at the pituitary level, which in its turn can stimulate the locally IGF-I production [6]. Since the protein levels in the skin would reflect a mixture between systemic and local production, without discriminating the IGF-I source, we analyzed IGF-I mRNA expression in the skin for the evaluation of local IGF-I production. As already observed in other non-hepatic tissues [3, 4], none of the members of the IGF-system signaling was compensatory increased in LI-IGF-I-/- mice with or without diabetes. Moreover, no difference was observed at the local IGF-I protein expression during wound healing between WT and LI-IGF-I-/- mice. In conclusion, the similar wound healing rate observed is not due to a compensatory increase in the local IGF-I system signaling.

The Mx-cre 31 interferon inducible model used for generating the LI-IGF-I-/- mice is not targeting just the hepatocytes, but also can alter IGF-I expression in leukocytes such as macrophages and other cells that are involved in the wound healing, as suggested by the reduction in IGF-1 expression observed in the spleen [3]. Therefore, we have analyzed IGF-I expression in different wound cell types over the time course of healing by analyzing the co-localized IGF-I expression in CD31-endothelial cells, in CD11b-positive leukocytes (granulocytes, monocytes, macrophages, and lymphocytes) and in keratinocytes. IGF-I levels decrease in the leukocytes from the wounds of LI-IGF-I-/- mice compared with WT mice at seven days post wounding, but not at wounding time or at the end of the healing process. No difference in IGF-I expression was observed in the other cell types analysed. The transient reduction in IGF-I expression in leukocytes either does not play a role for the healing process or does not reach a magnitude enough to modify the healing rate (as reflected by the same amount of total IGF-I protein levels in LI-IGF-I-/- and WT control).

An increase in the IGF-I bioavailability is another potential explanation for the preserved wound healing capacity in mice with inactivated liver-derived IGF-I. Approximately 70–80% of IGF-I in the circulation is maintained in the ternary complex which consists of IGF-I, IGFBP-3, and the acid labile subunit (ALS). A smaller, binary complex comprising IGF-I and serum IGFBPs (mainly IGFBP-3), comprises about 15–20% of the circulating pool while just 5%is free IGF-I which is directly bioavailable [16].In the more complex forms IGF-I has a longer half-life than the free IGF-I. An increase of the ALS levels concomitant with a decrease of IGFBP-3 has been reported in mice with liver-specific inactivation of the IGF-I gene [7], that will potentially modify the distribution of the IGF-I between the ternary and binary complexes and the free IGF-I pool.

Another explanation for the preserved wound healing potential in LI-IGF-I-/- mice could be the direct effect of the compensatory increase in GH secretion as GH is expressed in the skin [17]. This idea is supported by the observation that the wound healing rate is improved in animal models of diabetic wounds after local treatment with GH [18, 19]. Further evidence for this mechanism could be provided by studying the wound healing rate in bitransgenic mice carrying both liver-specific IGF-I deletion and GH deletion.

In our study we have also addressed the role of liver-derived IGF-I for the impaired wound healing rate in diabetes. The experimental data from the diabetic mice suggests that the lower levels of serum IGF-I registered both in patients with type 1 diabetes [20, 21] and in patients with insulin-dependent type 2 diabetes [22, 23] probably do not contribute to the impaired wound healing. This issue is of potential therapeutic importance suggesting that locally delivered IGF-I would be sufficient to improve wound healing in diabetes avoiding in this way the side effects that would be associated with systemic IGF-I therapy [24].

## Supporting information

S1 Fig Representative histological images (H&E) of wounds from LI-IGF-I-/- mice versus WT control. (TIF)

S2 Fig CD11b expression in the tissues during wound healing process. After immunohistochemical staining of CD11b in the tissues from wounding day (Day-0) and after 7 days (Day-7) and 14 days (Day-14) post wounding, the percentage of IGF-I positive cells were evaluated.
(TIF)
