# Supplementary figures and images for: Deficiency of liver-derived insulin-like growth factor-I (IGF-I) does not interfere with the skin wound healing rate
Source: PLoS One. 2018 Mar 13;13(3):e0193084. doi: 10.1371/journal.pone.0193084 (PMC5849293; doi:10.1371/journal.pone.0193084)

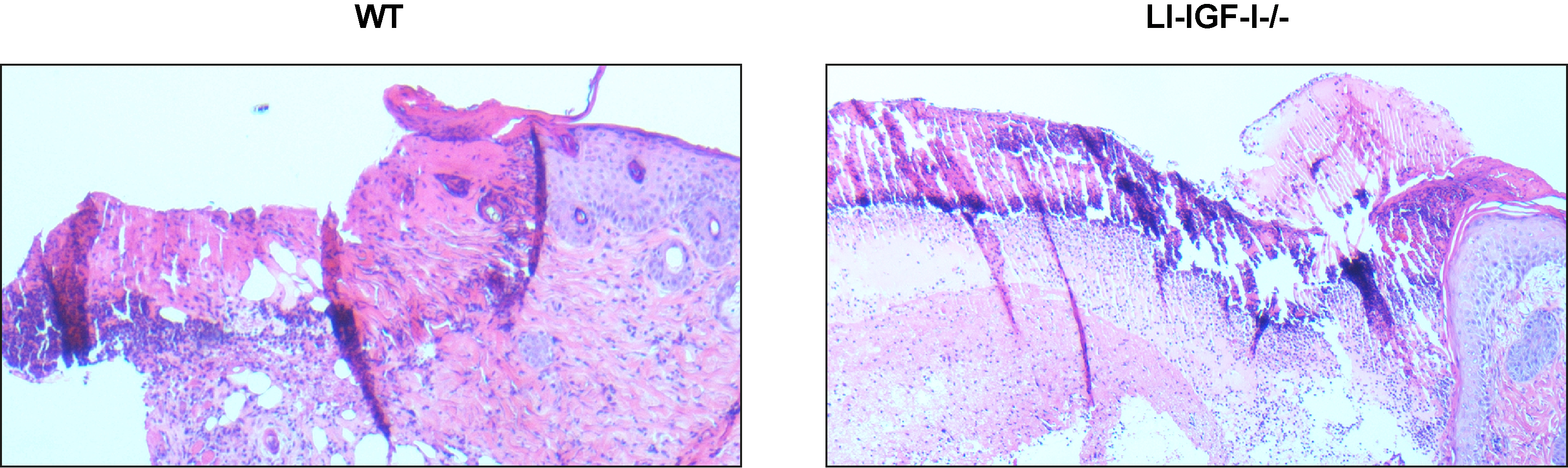

Supplement: S1 Fig — (TIF) [file pone.0193084.s001.tif]

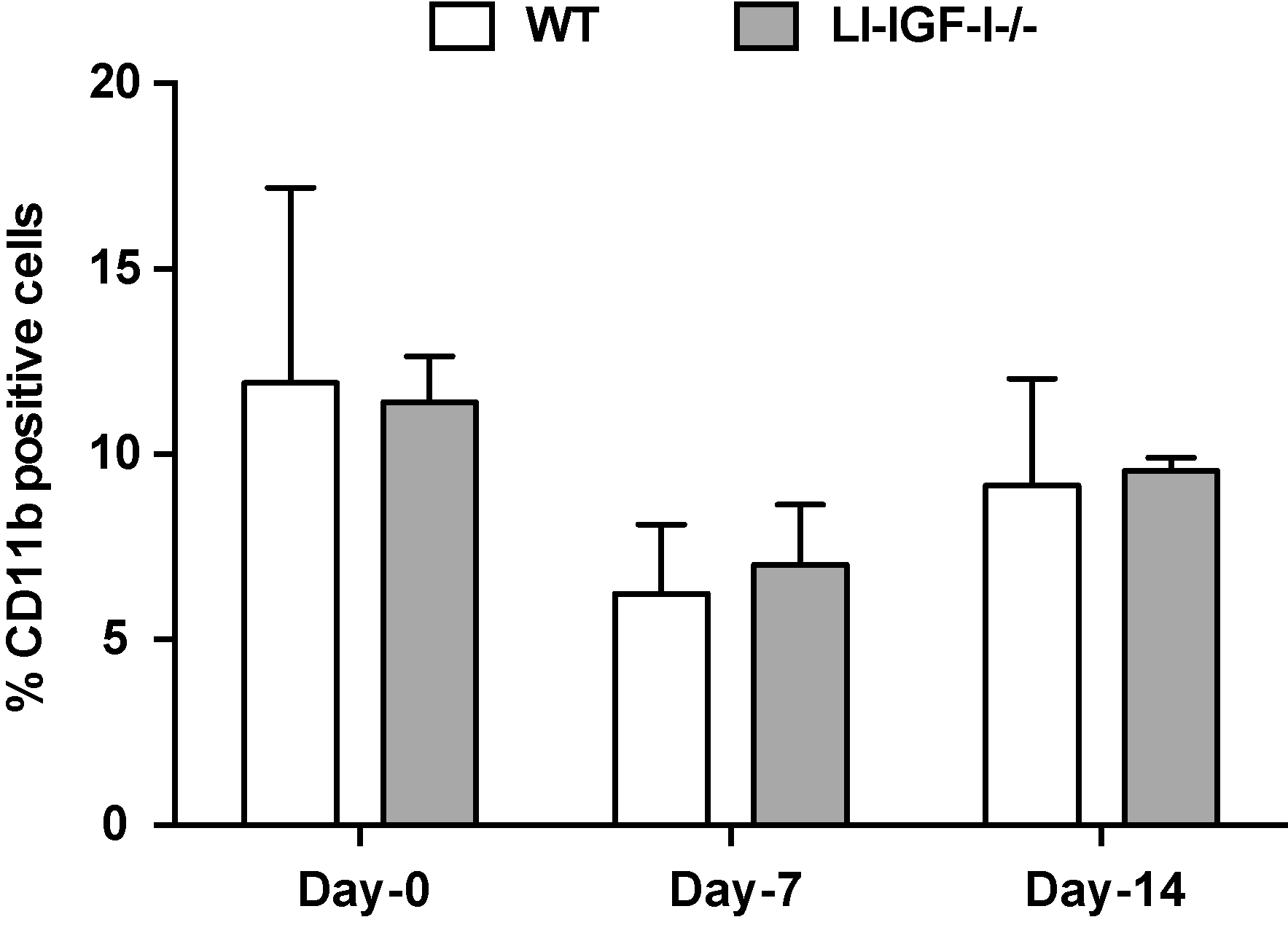

Supplement: S2 Fig — After immunohistochemical staining of CD11b in the tissues from wounding day (Day-0) and after 7 days (Day-7) and 14 days (Day-14) post wounding, the percentage of IGF-I positive cells were evaluated. (TIF) [file pone.0193084.s002.tif]
